# Evaluation of water, sanitation and hygiene program outcomes shows knowledge-behavior gaps in Coast Province, Kenya

**DOI:** 10.11604/pamj.2016.23.145.7546

**Published:** 2016-03-30

**Authors:** Michael Paul Schlegelmilch, Amyn Lakhani, Leslie Duncan Saunders, Gian Singh Jhangri

**Affiliations:** 1 School of Public Health, University of Alberta, Edmonton, Alberta, Canada; 2 Aga Khan University, East Africa

**Keywords:** Water, sanitation, hygiene, evaluation, knowledge, behavior, gap, coast

## Abstract

**Introduction:**

Water related diseases constitute a significant proportion of the burden of disease in Kenya. Water, sanitation and hygiene (WASH) programs are in operation nation-wide to address these challenges. This study evaluated the impact of the Sombeza Water and Sanitation Improvement Program (SWASIP) in Coast Province, Kenya.

**Methods:**

This study is a cluster randomized, follow-up evaluation that compared baseline (2007) to follow-up (2013) indicators from 250 households. Twenty-five villages were selected with probability proportional to size sampling, and ten households were selected randomly from each village. Follow-up data were collected by in-person interviews using pre-tested questionnaires, and analyzed to compare indicators collected at baseline. Cross-sectional results from the follow-up data were also reported.

**Results:**

Statistically significant improvements from baseline were observed in the proportions of respondents with latrine access at home, who washed their hands after defecation, who treated their household drinking water and the average time to collect water in the dry season. However, this study also observed significant decreases in the proportion of respondents who washed their hands before preparing their food, or feeding their children, and after attending to a child who has defecated. The analysis also revealed a knowledge-behavior gap in WASH behaviors.

**Conclusion:**

SWASIP contributed to improvements from baseline, but further progress still needs to be seen. The findings challenge the assumption that providing infrastructure and knowledge will result in behavior change. Further understanding of specific, non-knowledge predictors of WASH related behavior is needed.

## Introduction

It is estimated that 10% of the global burden of disease results from unsafe water, poor sanitation or inadequate hygiene [1]. Due to poor management of water resources and inadequate sanitation, the human consumption of unsafe water poses a major challenge to population health in many regions of the world [2]. The scope of these problems is broad and even though the etiologies of disease are varied, they are transmissible and thus, preventable [3]. Unfortunately, these diseases persist because 900 million people globally live without access to safe-water [1], and one billion people live without access to any type of sanitation facility whatsoever [3]. In Kenya, 17 million of the country's 40 million inhabitants do not have access to clean drinking water [4]. Water scarcity is becoming a more pressing concern as the population of Kenya is growing faster than infrastructure can be built for water and sanitation. The World Bank estimates that from 2011 to 2025, Kenya's per capita water consumption will drop from 630 to 235 cubic meters per person per year [4]. In the wake of the Millennium Development Goals (MDG), there are a number of programs operating that specifically target water and hygiene instability, yet many face sustainability challenges with infrastructure, continuity of funding and program policy support [5–7]. Sombeza Water and Sanitation Improvement Program (SWASIP), a multi-faceted water, sanitation and hygiene (WASH) intervention, was implemented between 2007 and 2010 predominantly in the district of Kinango, Coast Province, Kenya. SWASIP was a joint project between Aga Khan University, Department of Community Health in Mombasa and by the Coastal Rural Support Program, of Aga Khan Foundation, East Africa. SWASIP had three major program components. Firstly, the program constructed water and sanitation infrastructure in schools and communities such as roof water catchments, latrine blocks, hand hygiene stations, small farm reservoirs, public taps and community pipelines. Secondly, the program delivered health and hygiene promotion education to communities and schools, employing Community Led Total Sanitation (CLTS) methods, which have been adopted by over 60 countries worldwide as the primary means to improve sanitation in rural communities. CLTS aims to sensitize communities to the importance of sanitation and hygiene and eliminate open defecation [8]. Lastly, SWASIP constituted Water User Associations to manage and maintain the community WASH infrastructure. In this paper, we presented the results of a household survey, which was one component of an impact evaluation that was conducted in 2013 to assess the sustainability and impact at the household level of these WASH interventions.

## Methods

### Study Design

This study is a cluster randomized comparison study between baseline in 2007 and follow-up in 2013. We surveyed 250 households in the Kinango district of Coast Province, Kenya.

### Sample Size and Participant Selection

This study was designed to detect a 15% change from baseline on key indicators including latrine coverage, distance to water source, and hygiene behaviors (α=0.05, two-sided, and power=80%). Households were sampled by using probability proportional to size cluster sampling. The design effect of cluster sampling was calculated to be 1.27, based on the intra-cluster correlation coefficient of 0.03 from a WASH study in Nyanza Province, Kenya [9]. The required sample size was estimated to be 218 households. Twenty-five of the 67 villages in Kinango that were intervened by SWASIP were selected by probability proportional to size cluster sampling. A total of 250 households were selected, 10 households were randomly selected from each of these 25 villages. One participant from each selected household was interviewed. This person had to match the following inclusion criteria: had been residing in that household for more than 3 years, was older than 18 years old, and was the primary caregiver of the household.

### Data Collection

The survey tool combined relevant items from the USAID Hygiene Improvement Project [10], and the SWASIP tools used in 2007 for a baseline study. Behaviour change questions were modelled on the “Focus on Opportunity, Ability and Motivation” (FOAM) framework for hygiene and sanitation behaviour change [11, 12]. The survey tool was pre-tested in the neighbouring district of Msambweni, and assessed for feasibility, timeliness and accuracy of English-Swahili translations. Questions were also back translated to English and reviewed for accuracy. Enumerators with local knowledge of the project areas and experience with data collection were trained and hired to collect household data. Baseline data were retrieved from an unpublished baseline study conducted in 2007 by Aga Khan University, Department of Community Health, in Kinango, but prior to the initiation of SWASIP. Point estimates of mean time to water access and percentages of respondents performing specific WASH behaviours were reported, however, measures of variance were missing.

### Data Management and analysis

Data were entered into a data entry screen using EpiInfo 7 [13]. To minimize data entry errors, 50% of the data were re-checked for accuracy and were found to be accurate. Statistical analyses were done using STATA-12 [14]. Descriptive statistics were conducted on survey outcomes to report summary statistics. Eleven indicators were identified with sufficient baseline (2007) data to allow for direct comparisons with 2013 data (follow-up analysis). Two sided one-sample t-tests were conducted on these indicators to compare with baseline estimates. Results were expressed as mean ± 95% confidence interval (95% CI) for continuous variables and proportions (or percentages) ± 95% CI for dichotomous variables. Logistic regression analysis was used to find predictors of hand washing behavior, latrine ownership, and household drinking water treatment. Results were reported as odds ratio (OR) ± 95% CI. A p < 0.05 was considered for statistical significance.

### Ethics

Ethics approval was obtained for this study from the University of Alberta Research Ethics Office in Edmonton, Canada, and from the Aga Khan University Research Ethics Committee in Nairobi, Kenya. Informed verbal consent was obtained from study participants. Confidentiality was strictly maintained throughout data management, analysis and report writing.

## Results

### 2013 Participant Demographics

Survey respondents were predominantly 18-30 year-old primary caregivers, with low education levels. Forty-two percent (42%) of the total respondents reported having no education whatsoever and 62% of the total had less than Class 6. Two-thirds (68%) of respondents were employed as farmers or unemployed, while the remainder were either in small business or a working professional. When respondents were asked if they felt responsible for their own health, and the health of their family, 90% answered yes. Demographics for baseline respondents were unavailable.

### Follow-up analysis

Eleven indicators were compared with baseline data from 2007 (Table 1). All, except two of the comparisons, were statistically significant, however, not all of these significant findings indicated improvement over time. Water access for respondents improved most notably during dry season as respondents reported a reduction in the average time to collect water of 53.5 minutes which was a significant reduction (p<0.001) from the average time of 149.1 minutes, reported at baseline. Significant improvements were also seen in latrine coverage as baseline coverage increased 24% (p<0.001) from 19% to 43% of households indicating they had access to a latrine at home in 2013. The comparisons were made to assess the changes in hand hygiene behavior at five critical moments for hand hygiene [15]. Only hand hygiene practices after defection improved from 2007 to 2013 (63% to 73%, p=0.001). There was a decrease in the percentages of self-reported hand washing at the remaining four critical moments for hand hygiene behavior which are: before preparing food (-9%), before feeding children (-28%), before eating (-4%), and after attending to a child who has defecated (-37%).

**Table 1:** Comparison of WASH Related Indicators for the Follow-Up (2013) data compare to Baseline (2007) estimates

Household Level Indicator	Baseline Point Estimates	Follow-up Results	Change from Baseline to Follow-up (2007 to 2013)	
	Mean	Mean (95% CI)	Change (95% CI)	p-value
Time to collect water in wet season (min)	33.2	33.7 (29.0, 38.5)	0.5 (-4.2, 5.3)	0.826
Time to collect water in dry season (min)	149.1	95.6 (84.8, 106.5)	-53.5 (-64.3, -42.6)	<0.001
	Proportion	Proportion (95% CI)	Change (95% CI)	p-value
Treats household drinking water	0.41	0.48 (0.41, 0.54)	7% (0%, 13%)	0.038
Has access to a toilet facility at home	0.19	0.43 (0.37, 0.49)	24% (18%, 30%)	<0.001
Has access to an improved toilet facility at home	0.12	0.31 (0.25, 0.36)	19% (6%, 17%)	<0.001
Has a garbage pit	0.30	0.60 (0.54, 0.66)	30% (24%, 36%)	<0.001
Washes Hands:				
Before preparing food	0.37	0.28 (0.22, 0.34)	-9% (-15%, -0.4%)	0.003
Before feeding children	0.48	0.20 (0.15, 0.24)	-28% (-33%, -24%)	<0.001
Before eating	0.93	0.89 (0.86, 0.93)	-4% (-8%, 0%)	0.056
After defecation	0.63	0.73 (0.68, 0.79)	10% (4%, 15%)	0.001
After cleaning a child who has defecated	0.55	0.18 (0.14, 0.23)	-37% (-41%, -32%)	<0.001

### Knowledge behavior gap

**Figure 1:**
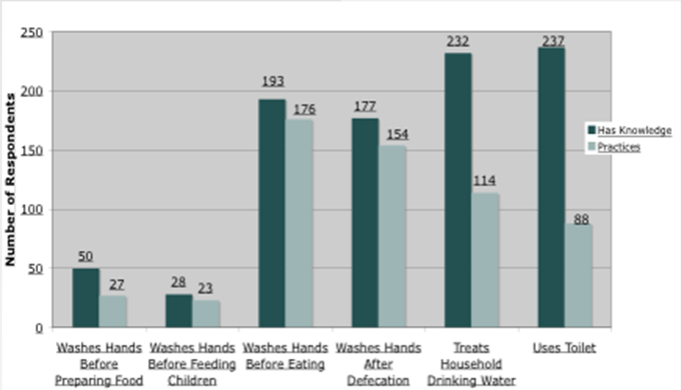
Bar graph of knowledge-behavior gap on six WASH indicators from respondents in 2013

Six indicators covering hand hygiene, water treatment and toilet use were selected for a knowledge-behavior gap investigation. Differences between knowledge and behavior were observed, at varying degrees, in each of the six indicators and are displayed in Figure 1, with the dark bars showing respondents who had knowledge of health behaviors and light bars showing respondents who actually practiced that health behavior. The two largest knowledge-behavior gaps were observed in drinking water treatment and toilet use. Of the 232 respondents who said that treating their household drinking water will help keep their family healthy, 114 (49%) actually treated their drinking water. In terms of toilet use, a larger gap was seen. With latrine use, 237 respondents reported that they understood the benefits of defecating in a toilet facility, however, only 88 (37%) of those respondents used a toilet facility themselves.

### Barriers to household WASH behaviors

As reported in Table 2, the most commonly reported barrier to safely treating household drinking water was that supplies could not be procured (44%), followed closely by 36% of respondents stating that they could not afford water treatment supplies. Together, this can be taken to mean that 80% of respondents faced economic challenges to treating their drinking water. As was seen with barriers to treating household drinking water, 84% of respondents listed affordability of supplies, in this case soap, as the primary inhibiting factor to practicing hand washing with soap. However, 90% of respondents used soap when hand washing. We defined soap use as an answer of either “always”, or “sometimes”to the question, “When you wash your hands for any reason, do you use soap”. In the follow-up analysis, overall latrine accessibility at home did improve from baseline (19% to 43%), yet 19% of respondents who had access to a latrine at home, and 75% of respondents overall, reported that at least one member of their household still practiced open defecation. The most common explanation (55%) for practicing open defecation among respondents was that the latrine was too far away for convenient use, even if it was accessible from home.

**Table 2:** Common reasons given to explain lapses in healthy WASH behavior

Indicator	n(%)
**Water Management**	
Most common reasons for not treating water (n=162)	
1) No supplies	71 (43.8)
2) No money	59 (36.4)
3) Water source doesn't need to be treated	33 (20.3)
	
**Personal Hygiene**	
Most common reasons for not washing hands (n=187)	
1) Can't afford soap	157 (84.0)
2) Don't have time	34 (18.2)
3) Don't see the need	11 ( 5.9)
	
**Sanitation**	
Most common reasons for not having a toilet facility at home (n=232)	
1) Haven't had time to build	60 (25.9)
2) High cost of construction	35 (15.1)
3) Pit has collapsed	20 ( 8.6)
	
Most common reasons for not using a toilet facility (n=159)	
1) A latrine is too far away	87 (54.7)
2) Latrine has collapsed	20 (12.6)
3) I prefer the bush	20 (12.6)

### Predictors of household WASH behaviors

The logistic regression analysis results of outcomes hand washing with soap and latrine ownership are shown in Table 3. Controlling for education and employment status, respondents who indicated that they felt a responsibility for their own health had the greatest odds (OR=3.51, p=0.017) of washing their hands with soap compared to respondents who felt no responsibility for health, both education and employment status were at borderline significance (p=0.072 and 0.051, respectively). The odds of latrine ownership were significantly predicted by education (OR=2.55, p<0.001), when controlling for employment and felt responsibility, but employment status and felt responsibility were non-significant for this outcome. Neither education nor occupation nor a felt responsibility for their own health were found to be significant predictors of household drinking water treatment (results not shown).

**Table 3:** Odds Ratios (OR) and 95% confidence interval (95% CI) for Predictors of Hand Washing Behavior and Latrine Ownership

	Univariate Analysis		Multivariable Analysis	
Variables	OR (95% CI)	p-value	OR (95% CI)	p-value
**Outcome: Washes hands with Soap**				
Education of Class 6 or above	3.69 (1.23, 11.08)	0.020	2.81 (0.91, 8.64)	0.072
Businessman or other professional	4.04 (1.18, 13.89)	0.027	3.51 (0.997, 12.4)	0.051
Feels Responsibility for health	4.25 (1.57, 11.53)	0.005	3.51 (1.25, 9.84)	0.017
**Outcome: Owns a Latrine**				
Education of Class 6 or above	2.62 (1.55, 4.44)	<0.001	2.55 (1.47, 4.43)	<0.001
Businessman or other professional	1.22 (0.71, 2.08)	0.472	1.11 (0.63, 1.98)	0.712
Feels Responsibility for health	1.27 (0.53, 3.03)	0.589	0.99 (0.40, 2.41)	0.987

## Discussion

The improvements in water access and sanitation facility coverage were significant and are a testament to successful programming. There is a known, complementary health benefit to communities when latrine coverage and water consumption are improved concurrently and these benefits will likely be appreciated [16]. It should be noted, however, that the overall levels of latrine coverage are still below 50% of households. Hand hygiene practices at five critical moments did not improve, except for those who washed their hands after defecating. This may be due to a particular educational focus on hygiene with latrine use, but no specific indicators from this evaluation can offer a definitive explanation of this finding. The assumption that health education will result in behavior change has been a point of contention in public health since the 1980's and likewise, providing infrastructure or equipment does not ensure it will be used [17, 18]. Though the SWASIP program did deliver hygiene education and infrastructure successfully [19], challenges with hygienic practices remain. This study shows a knowledge behavior gap in hand hygiene and latrine use that suggests there are other barriers to safe WASH practices, beyond a lack of knowledge. Other research in Coast Province, Kenya found that 71% of respondents understood the importance of hand washing after defecation while 31% actually did so and similar knowledge-behaviors gaps with hand hygiene have been reported in Bangladesh [6, 11]. Graves et al. found that school hygiene programs in Western Kenya suffer from supply stock outs and unaffordable hygiene supplies. These complaints are also the two most common barriers to household hygiene reported by our respondents [20].

Our findings suggest that even when education is combined with infrastructure, sustained and consistent latrine use is not completely achieved. This study found that while 43% of respondents had access to a latrine at home, 19% of them reported that at least one member of their family still practiced open defecation. The most common reason respondents gave to explain open defecation practices was that although a latrine was available, it was too far away. This highlights an important distinction between latrines that are accessible and ones that are accessible enough to be consistently used. We can theorize that a respondent may indicate they understand the importance of using a latrine, have access to a latrine, perhaps a neighbour's, and yet choose not to use it because it is less convenient or uncomfortable. Similar results have been documented in Tanzania with mothers reporting that, even though they understood the benefits, safer WASH practices can be too impractical from them to adopt [21]. Another theory to explain sub-optimal latrine use posits that cultural taboo influences latrine use. In Kilifi, a neighbouring district to where this study was conducted, some residents believe that a man's feces should never mix with his daughter-in-law's or that a person's feces can be used in witchcraft to bewitch him [22]. Our study did not find evidence that could support or refute this theory. None of our respondents mentioned taboo as a barrier to latrine use, however there may a social desirability bias to answering questions on latrine use in a socially acceptable way. This collection bias may have also artificially inflated the proportion of respondents reporting that they practice good hand hygiene and use a latrine, which has been described by other researchers working in South Asia [23]. In addition to the collection bias described above, this study was limited by baseline data that were incomplete and variance statistics of mean point estimates could not be utilized in the analysis but were assumed to be equal at baseline and follow up. The design effect was excluded from analysis. However, this would only increase the confidence intervals reported and would not change the point estimates of proportion. Considering the low p-values in the follow-up analysis, including the design effect in analysis would very likely not affect the conclusions.

## Conclusion

Significant improvements from baseline were observed, yet overall levels of latrine coverage are still low. This is likely a symptom of a successful project that was terminated before larger gains could be realized as self-sustaining behavior change may take longer commitments than a three-year program. Healthy WASH practices are still hindered, predominantly, by non-knowledge barriers such as convenience and financial insecurity. There are two recommendations for further practice. The first is to reinstate the successful health and hygiene promotion interventions to continue progress with increasing latrine coverage and healthy WASH practices. Along with this, it is recommended that funders consider this needed longevity when describing funding terms. Secondly, future programming must not rely on an unverified assumption that providing knowledge and infrastructure, even together, will result in changes in hygiene or sanitation behaviors. The socio-cultural context in which WASH decisions and behaviors are operating is complex and intermingled. It would be prudent to first understand and describe the non-knowledge predictors of WASH practices in a community when conceptualizing future WASH programs for implementation.

### What is known about this topic

- Unsafe water and poor sanitation are significant contributors to global morbidity and mortality.
- Kenya is experiencing water scarcity and low latrine coverage with population needs outgrowing infrastructure support.
- Challenges of sustainability with WASH infrastructure exist due to inconsistent funding and policy support.

### What this study adds

- Short term or intermittent funding for WASH infrastructure precludes its safe and reliable functioning.
- There is a knowledge behavior gap with WASH practices, likely due severe financial constraints, inconvenience and to a lack of felt responsibility for health.
- It is unfounded to assume that providing WASH infrastructure and education, even together, will affect practices. The socio-cultural context needs to be considered when designing health behavior change programming.
